# In vivo dual-plane 3-photon microscopy: spanning the depth of the mouse neocortex

**DOI:** 10.1364/BOE.544383

**Published:** 2024-11-26

**Authors:** Matilda Cloves, Troy W. Margrie

**Affiliations:** The Sainsbury Wellcome Centre for Circuits and Behaviour, University College London, 25 Howland Street, W1T 4JG, London, United Kingdom

## Abstract

Cortical computations arise from patterns of neuronal activity that span across all cortical layers and cell types. Three-photon excitation has extended the depth limit of *in vivo* imaging within the mouse brain to encompass all cortical layers. However, simultaneous three-photon imaging throughout cortical layers has yet to be demonstrated. Here, we combine non-unity magnification remote focusing with adaptive optics to achieve single-cell resolution imaging from two temporally multiplexed planes separated by up to 600 µm. This approach enables the simultaneous acquisition of neuronal activity from genetically defined cell types in any pair of cortical layers across the mouse neocortical column.

## 1. Introduction

Two-photon (2p) laser scanning microscopy has attained widespread popularity within neuroscience research by virtue of its confinement of excitation within scattering tissue, thereby providing images at micrometre resolution deep within living, intact brains [1–3]. When used to image fluorescent calcium indicators, 2p microscopy has enabled activity measurements from progressively larger numbers of individually identifiable and genetically defined neurons in awake, behaving animals [4–13]. Accordingly, the advents of 2p microscopy and genetically encoded calcium indicators have given rise to many landmark discoveries across systems neuroscience, particularly regarding our understanding of cortical computations in mice [14–17].

A cardinal component of cortical processing comprises the interactions between neurons residing in different cortical layers [18–21]. Similarly, cortico-cortical feedback – thought to subserve cortical processes such as sensory perception, attention and prediction [22–24] – is mediated by inter-areal projections with layer-specific termination patterns which engage local inhibitory circuitry to orchestrate layer-specific patterns of excitation, inhibition and disinhibition across multiple cell types [25–31], and has recently been demonstrated to be dominated by Layer 6 (L6) [32]. Thus, pursuing a full understanding of any cortical computation requires the capacity for imaging neuronal activity occurring simultaneously across cortical layers, including the deepest layer, L6.

Accumulating evidence suggests L6 occupies a prominent position in cortico-cortical communication [32,33]. L6 has recently been found to provide the main source of feedback projections to primary sensory and motor areas, with the hierarchical ranking of an area correlating with the proportion of its feedback projections arising from L6 relative to L2/3 [32]. Investigating the functional implications of L6 dominance over cortical feedback would be well informed by simultaneous imaging of L6 and L2/3 feedback projection neurons. Furthermore, in primary sensory areas, feedback projection axons and neuromodulatory fibres often ramify in both L1 and L5/6 [27,34–37]. How the signals carried by deep inputs compare to those carried by their superficial counterparts would be revealed by dual-layer axon imaging. Altogether, these open questions suggest the need for an imaging approach able to record from neurons in deep (L6) and superficial (L2/3) layers simultaneously.

Multi-layer calcium imaging has been achieved by an array of refocusing strategies over the last two decades, from physically displacing the imaging objective [38] to changing the vergence angle of the beam entering the objective via an electrically tunable lens [39], spatial light modulator [40], second objective [41,42] or concave mirrors [13]. However, these approaches have been constrained by the ∼600 µm depth limit of 2p excitation, rendering them blind to the activity occurring in deeper sections of L5 and all of L6 [43]. To push against this constraint, a recent application of hybridised 2p-3p excitation leveraged the longer excitation wavelengths and improved depth penetration of 3p imaging to achieve near-simultaneous recordings across the entire cortical depth [44]. However, the resulting two-beam optical system is complex and wholly specialised, involving two separate scan engines to handle the two temporally multiplexed excitation beams produced by a laser source designed specifically for 2p-3p imaging (White Dwarf Hybrid, Class 5 Photonics). Further, while recording simultaneously across all cortical layers provides valuable data necessary to attain a full understanding of cortical function, several questions still remain regarding the roles played by neurons in L6 which could be investigated with only two imaging planes, one deep and one superficial. These experiments would be best served by dual-plane 3p calcium imaging, an approach which accesses depths beyond the limitations of 2p imaging while avoiding the quagmire of hybridising 2 and 3p excitation.

The most straightforward acquisition strategy for dual-plane 3p calcium imaging is temporal multiplexing. The relatively slow laser pulse repetition rates (often ≤ 1 MHz) used for 3p imaging mean that frame rates for even small (∼150 µm^2^) field of view (FOV) acquisitions are limited to ∼7 Hz for 512 x 512 images sampled with 1 pulse-per-pixel. To an extent, frame rates can be improved by reducing the number of pixels per frame, although lowering resolution risks degrading calcium signals as fewer pixels are devoted to each cell. Broadly, the limitation placed on sampling frequency by laser repetition rate renders frame-wise refocusing systems too slow to achieve the volume rates required to adequately sample the temporal dynamics of calcium indicators. Indeed, dual-plane 3p calcium imaging has recently been demonstrated with two temporally multiplexed beams, one of which was refocused by a remote objective to achieve 50 µm axial separation of the two planes [45]. However, larger axial separations of imaging planes are required for experiments dissecting the interlaminar circuits onto and from cortical L6, particularly those which span the supragranular layers. This requirement would only be satisfied by a refocusing system capable of producing 800 µm axial separation between two imaging planes while maintaining single-cell resolution *in vivo*. The dual-plane 3p imaging technique presented here is designed for this purpose and allows two simultaneous, single-cell resolution imaging planes to be placed in any pair of cortical layers.

## 2. Optical design

**Fig. 1.**
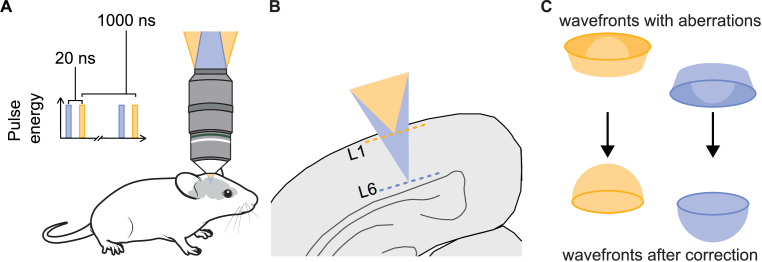
Illustrations of the aim of the system. **(A)** Two temporally multiplexed 1 MHz beams were used to image the neocortex of awake, behaving mice. **(B)** The two beams were independently defocused to image the most superficial and deepest cortical layers. **(C)** The wavefronts of the two beams were independently corrected for system-induced aberrations (examples of spherical aberrations are shown), leaving behind only the defocus wavefront.

The principal aim of this system design was to produce two 3p imaging planes which could be separated by the greatest possible axial distance whilst maintaining single-cell resolution. This was achieved through the independent remote focusing and system-induced aberration correction of two temporally multiplexed beams (Fig. 1).

**Fig. 2.**
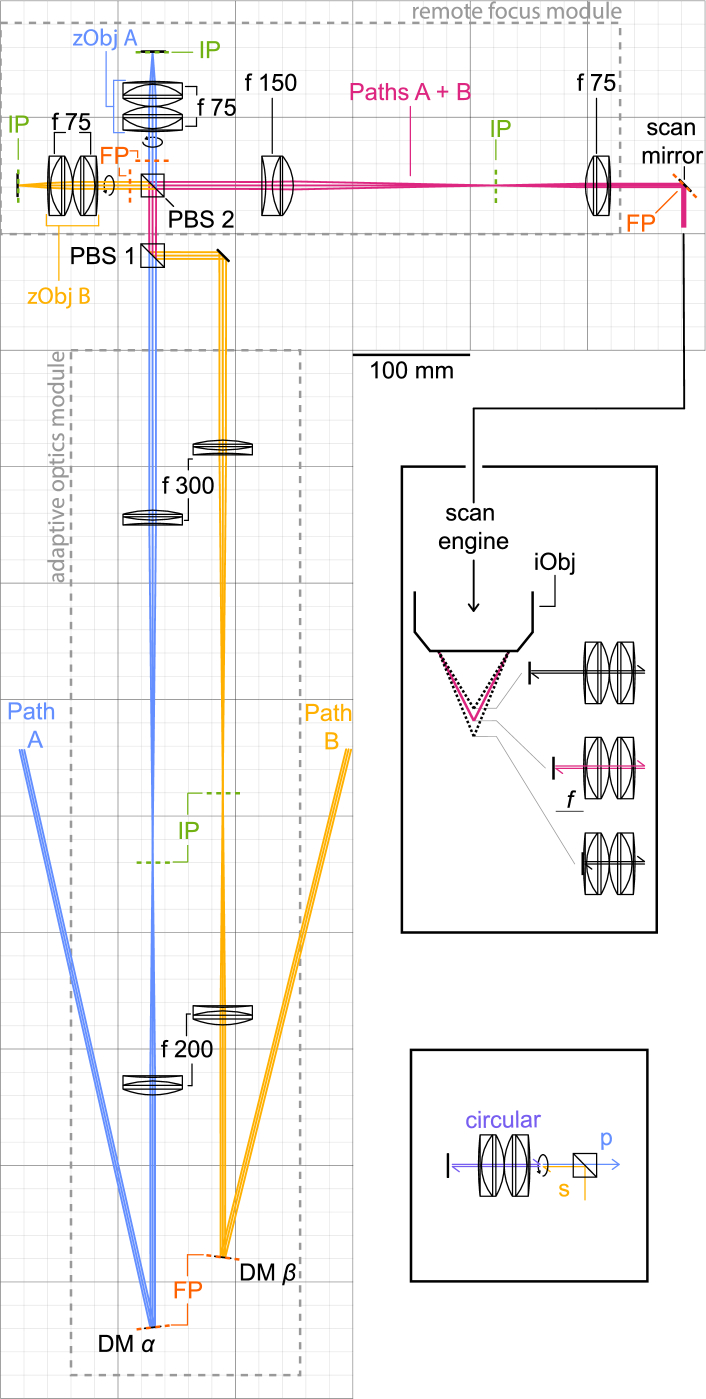
Schematic of the entire system. The non-delayed beam (blue) follows path A. The surface of DM *α* is conjugated with the back focal plane of the zObj and the surface of the first scan mirror. These conjugate planes are highlighted as Fourier plane (FP). The positions of the planes conjugate to the image plane (IP) are also highlighted. Path A starts as p-polarised and therefore passes through PBS cubes 1 and 2 before a double pass through a QWP (represented by a circular arrow) returns the beam as s-polarised to reflect off PBS cube 2. The delayed beam (yellow) follows path B which begins as s-polarised and comprises a configuration of lenses and distances identical to those in path A. The inset to the middle-right of the schematic shows both beams focused at the native focal plane of the iObj (pink) in accordance with the zObj configuration shown in the schematic. In black, positive and negative focal shifts are shown, labelled with their associated z-mirror shift. The inset to the bottom-right of the schematic shows the double pass through zObj B with the polarisation labelled.

A 1300 nm 1 MHz excitation beam was generated by an optical parametric amplifier (Opera-f, Coherent) pumped by a 60 W 1035 nm beam (Monaco, Coherent). The beam was attenuated with a half wave plate (HWP) and polarising beam splitter (PBS) cube pair before entering a single prism pulse compressor [46]. At the output of the amplifier, the pulse width was measured by an autocorrelator (Carpe IR, APE) to be 57 fs. The path length of the compressor was set to achieve a 59 fs pulse width under the imaging objective (iObj). After the pulse compressor, the beam was expanded to 4 mm 1/*e*^2^ diameter and then split by a PBS cube into two beams. The relative power of the two beams was adjusted by a HWP immediately before the PBS cube. The s-polarised beam was then delayed by ∼20 ns relative to the p-polarised beam by a fixed delay line with a 6 m path length, shielded from air currents. After the delay line, the two beams entered identical adaptive optics (AO) and remote focus systems before reuniting and entering the microscope (Fig. 2).

The AO system was replicated from a previously published design for a sensorless AO module [47]. First, the beam was reflected off a segmented deformable mirror (Hex-111-DM, Boston Micromachines) at a shallow angle. The DM surface was conjugated to the back focal plane of a remote z-scan objective (zObj) and magnified by 1.5× by a pair of achromatic doublets (ACT508-200-C and ACT508-300-C, Thorlabs).

The remote focus unit was replicated with minor alterations from a previously published design for non-unity magnification remote focusing [42]. The zObj comprised two achromatic doublets (AC508-75-C, Thorlabs) arranged in a Plössl design. Just before the zObj were a PBS cube and quarter-wave plate (QWP). After the beam was focused by the zObj, a z-mirror reflected the beam back for a second pass through the zObj, QWP and PBS cube.

Since the beam was circularly polarised by the first QWP pass, it emerged after the second QWP pass with a polarisation orthogonal to that with which it started. The non-delayed – initially p-polarised – path emerged from the remote focus unit as s-polarised and was reflected within the PBS cube, while the delayed – initially s-polarised – path emerged as p-polarised and passed through the same PBS cube. Thus, the two paths were reunited.

The back focal planes of the zObjs were conjugated to the scan mirrors and demagnified 0.5× by a pair of achromatic doublets (AC508-150-C and AC508-75-C, Thorlabs). The scan mirrors were conjugated to each other and to the objective (XLPLN25XWMP2, NA 1.05, 25×, Olympus) by the afocal relays and scan telescope design of the Trepan2p (Twin Region, Panoramic 2-photon) microscope [11]. The beam underfilled the back aperture of the iObj by 80 % to produce an effective numerical aperture (NA) of ∼0.7.

**Fig. 3.**
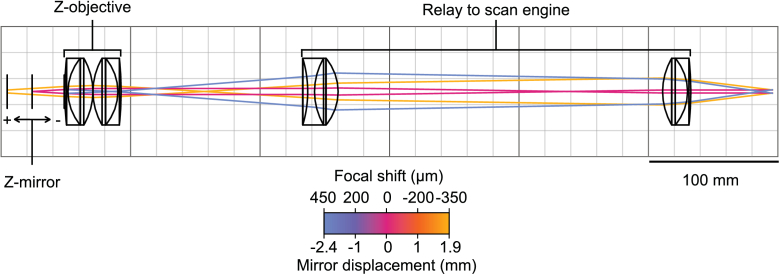
Detailed schematic of the remote focus module. Three configurations of the remote focus module and scan engine relay are shown, each with different z-mirror displacements which produce different focal shifts after the imaging objective. The colour of the rays drawn corresponds to the mirror displacement/focal shift pair shown on the colour bar. Rays are drawn from the surface of the z-mirror to the surface of the scan mirror and indicate the outer shape.

The difference in focal length between the zObj and iObj, alongside the difference in beam diameter entering the back apertures of these objectives, determines the ratio between the size of a z-mirror displacement and the iObj focal shift it produces (Fig. 3). The ratio produced by this system is ∼5.4 mm per 100 µm of focal shift, three times weaker than the ratio of 1.8 mm per 100 µm published by Rupprecht and colleagues [42]. This disparity is chiefly due to our relatively shorter iObj focal length. Compensating for this difference would require increasing the demagnification of the lens pair conjugating the zObj to the scan mirrors and thereby increase the footprint of the remote focus module. However, because the z-mirrors remain static during acquisitions rather than scanning through multiple displacements, we were free to accept a relatively weak ratio. Further, we were able to increase the focal length of the zObj by converting to a 2-inch Plössl lens in an effort to reduce the introduction of aberrations at larger focal shifts.

Emitted green fluorescence was separated from the excitation path by a dichroic mirror (T700lp-3p, Chroma), bandpass filtered (FF01-510/84-25), and then detected by a GaAsP photomultiplier tube (PMT, H16201P-40, Hamamatsu). The output of the PMT was amplified by a high-speed current amplifier (DHCPA-100, Femto).

When aberration correction was required, software carrying out an ’AO routine’ [47] adjusted the actuators of the DM to find and then apply a corrective wavefront. During this routine, the beam was parked at the centre of a fluorescent bead. While half of the DM segments remained fixed to form a reference focus, the actuators of the remaining segments were modulated at distinct frequencies. The Fourier transform of the fluorescence signal recorded during these modulations was used to find the position for each actuator which produced the strongest interference with the reference focus. This group of segments then remained stationary while the other half were adjusted in the same way.

While running the AO routine, the amplifier was set to 220 kHz bandwidth, $10^{7}$ V/A. While imaging temporally multiplexed planes, the amplifier was set to 80 MHz bandwidth, $10^{3}$ V/A. In order to distinguish fluorescence signals arising from the two temporally multiplexed beams, the sample clock of the data acquisition card was synchronised to the sync output of the Monaco laser. Two temporal filters (11 ns wide, separated by 8 ns) were placed over the PMT signal in ScanImage (2022.1.0, MBF Bioscience) to create two virtual channels synchronised with the excitation pulses. Images formed from delayed pulses were not qualitatively different in stability compared with those formed from non-delayed pulses.

## 3. Results

### 3.1. Resolution measurements

#### 3.1.1. Axial resolution

Axial resolution across focal shift was used as the chief performance metric of this system. In a non-unity magnification remote focus system, larger focal shifts introduce progressively larger spherical aberrations, the primary consequence of which is the degradation of axial resolution [41]. If single-cell resolution imaging in mouse cortex is to be maintained, the minimum axial resolution required of the multiphoton excitation point spread function (PSF) is considered to lie between 7 - 15 µm [44,48]. Since the scan mirrors were conjugated to each other and to the back focal plane of the zObj, we observed no change in FOV size across focal shift.

We measured the axial PSF as the full width half maximum (FWHM) of a Gaussian fit of the axial intensity profile of a sub-resolution fluorescent bead (either 5 or 1 µm in diameter depending on the expected PSF). At a range of focal shifts, 5 non-corrected and 5 aberration-corrected measurements were taken from 10 different beads. Aberration correction was achieved after executing the AO routine published by Rodríguez and colleagues [47] on the fluorescence signal acquired from pointing the excitation beam at the centre of the bead. The correction map for each focal shift was saved.

**Fig. 4.**
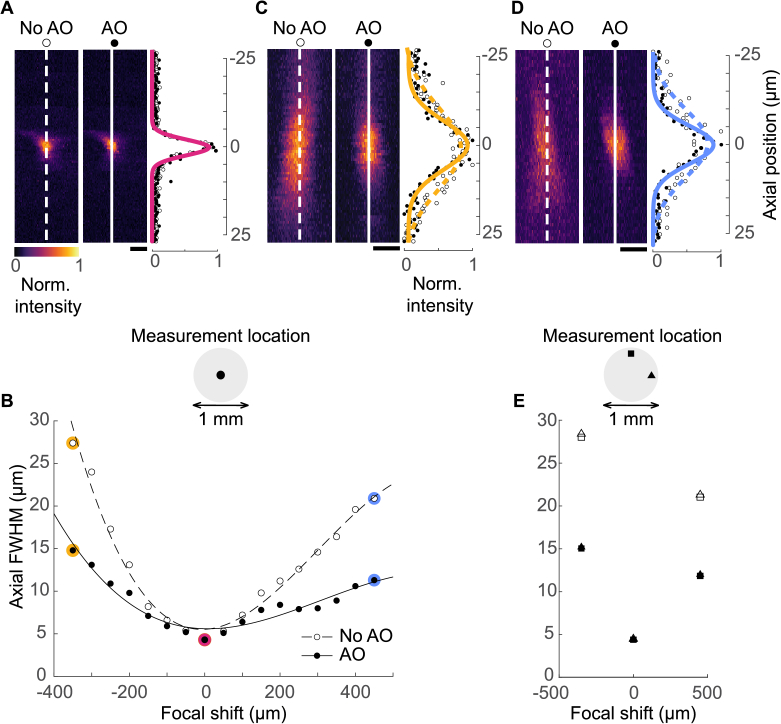
Axial resolution measurements across focal shifts with and without aberration correction. **(A)** Example YZ projections of a 1 µm bead taken at 0 focal shift and corresponding axial intensity projections with Gaussian fits. Scale bar is 1 µm in Y. Open markers indicate non-corrected measurements, filled markers indicate aberration-corrected measurements. **(B)** Axial FWHM as a function of focal shift. Each marker is the mean of measurements from 5 different beads taken at the centre of the FOV. The markers highlighted in magenta, yellow and blue indicate the focal shifts for which panels A, C and D serve as examples. **(C)** Example YZ projections of a 5 µm bead taken at - 350 µm focal shift and corresponding axial intensity projections with Gaussian fits. Scale bar is 5 µm in Y. **(D)** Example YZ projections of a 5 µm bead taken at + 450 µm focal shift and corresponding axial intensity projections with Gaussian fits. Scale bar is 5 µm in Y. **(E)** Measurements taken at the X-edge (triangles) and Y-edge (squares) of the FOV. Open markers indicate non-corrected measurements, filled markers indicate aberration-corrected measurements.

At the native focal plane of iObj (0 focal shift), the axial intensity profiles of the non-corrected (no AO) and aberration-corrected (AO) image stacks were entirely overlapping (example shown in Fig. 4(A)). Both the mean non-corrected axial FWHM and the mean aberration-corrected axial FWHM were 4.3 µm (Fig. 4(B)).

Negative focal shifts (toward iObj) result in a reduction of the effective NA of the iObj and therefore produce a steeper slope of axial FWHM expansion than positive focal shifts (away from iObj) which result in increases in the effective iObj NA. The slopes of the aberration-corrected measurements remain asymmetrical as the change in effective NA is independent of the wavefront shape (Fig. 4(B)).

At - 350 µm focal shift, the degradation of axial resolution by focal shift was apparent: the axial intensity profiles of the non-corrected image stacks were considerably wider than those observed at 0 focal shift and were made sharper with aberration correction (example shown in Fig. 4(C)). The mean non-corrected FWHM was 27.4 µm, while the mean aberration-corrected FWHM was 14.8 µm (Fig. 4(B)). This marks the limit of available negative shift while maintaining single-cell resolution.

Since positive focal shifts showed less degradation of resolution, they were instead limited by the lens diameters used in afocal relays of the scan engine. Shifts further than + 450 µm were associated with a loss of average power under the iObj as the beam began to clip. At + 450 µm focal shift, the axial intensity profiles of the non-corrected image stacks were sharper than those observed at - 350 µm focal shift and were made sharper still with aberration correction (example shown in Fig. 4(D)). Here, the mean non-corrected FWHM was 20.9 µm, while the mean aberration-corrected FWHM was 11.3 µm (Fig. 4(B)).

The above measurements were taken at the centre of the FOV of the microscope. Measurements were repeated at the X and Y edges (540 µm from centre) of the FOV for the positive and negative limits of focal shift as well as 0 focal shift (Fig. 4(E), 4F). The mean values obtained for non-corrected and aberration-corrected image stacks at the FOV edges were similar to those obtained at the FOV centre.

#### 3.1.2. Radial resolution

Radial resolution also suffers with focal shift, albeit to a lesser extent than axial. We repeated the same measurement regimen as before, recording the FWHM of the Lorentzian fit of a radial intensity profile of either a 1 or 0.5 µm fluorescent bead depending on the expected resolution.

**Fig. 5.**
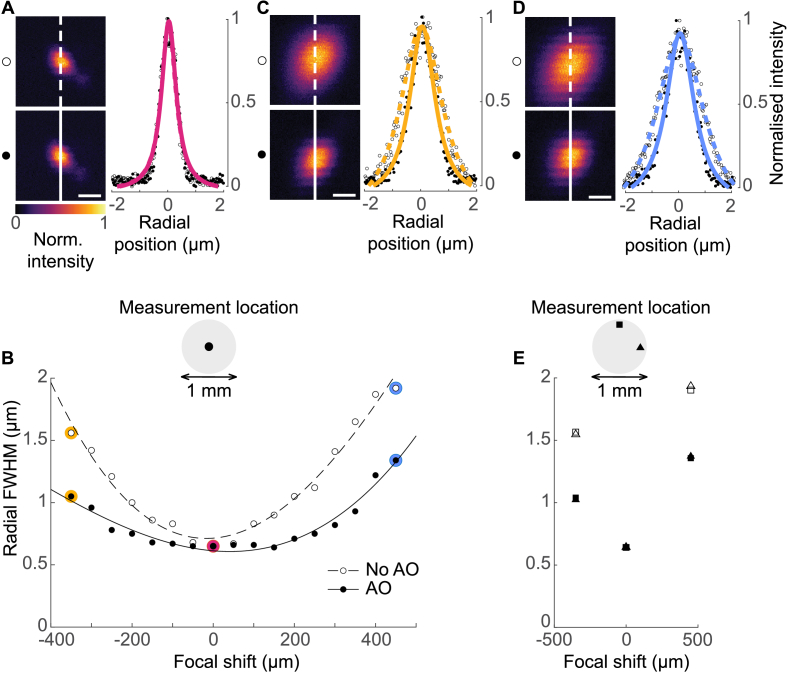
Radial resolution measurements across focal shifts with and without aberration correction. **(A)** Example images of a 0.5 µm bead taken at 0 focal shift and corresponding radial intensity projections with Lorentzian fits. Scale bar is 1 µm. Open markers indicate non-corrected measurements, filled markers indicate aberration-corrected measurements. **(B)** Radial FWHM as a function of focal shift. Each marker is the mean of measurements from 5 beads taken at the centre of the FOV. The markers highlighted in magenta, yellow and blue indicate the focal shifts for which panels A, C and D serve as examples. **(C)** Example images of a 1 µm bead taken at - 350 µm focal shift and corresponding radial intensity projections with Lorentzian fits. Scale bar is 1 µm. **(D)** Example images of a 1 µm bead taken at + 450 µm focal shift and corresponding radial intensity projections with Lorentzian fits. Scale bar is 1 µm. **(E)** Measurements taken at the X-edge (triangles) and Y-edge (squares) of the FOV. Open markers indicate non-corrected measurements, filled markers indicate aberration-corrected measurements.

As with axial resolution, at 0 focal shift the radial intensity profiles of the non-corrected and aberration-corrected images were entirely overlapping (example shown in Fig. 5(A)). Both the mean non-corrected radial FWHM and the mean aberration-corrected radial FWHM were 0.65 µm (Fig. 5(B)).

However, unlike with axial resolution, focal shift degraded radial resolution symmetrically (Fig. 5(B)). This was expected given that radial resolution is much less sensitive to changes in iObj NA than axial resolution is [49].

At - 350 µm shift, the radial intensity profiles of the non-corrected images were again considerably wider than those observed at 0 focal shift and were again made sharper with aberration correction (Fig. 5(C)). The mean non-corrected radial FWHM was 1.56 µm, which was improved with aberration correction to 1.05 µm (Fig. 5(B)). Given the symmetry of radial resolution degradation with focal shift, at + 450 µm focal shift, the radial intensity profiles of the non-corrected images were wider than those observed at - 350 µm focal shift and again were made sharper with aberration correction (Fig. 5(D)). Here, the mean non-corrected radial FWHM was 1.92 µm, which was improved with aberration correction to 1.34 µm (Fig. 5(B)).

Thus, between - 350 µm and + 450 µm focal shift, all axial PSFs recorded were < 15 µm while all radial PSFs recorded were < 1.4 µm, meaning single-cell resolution imaging could be preserved over 800 µm of axial separation.

### 3.2. Calcium imaging of spontaneous activity in mouse primary visual cortex

**Fig. 6.**
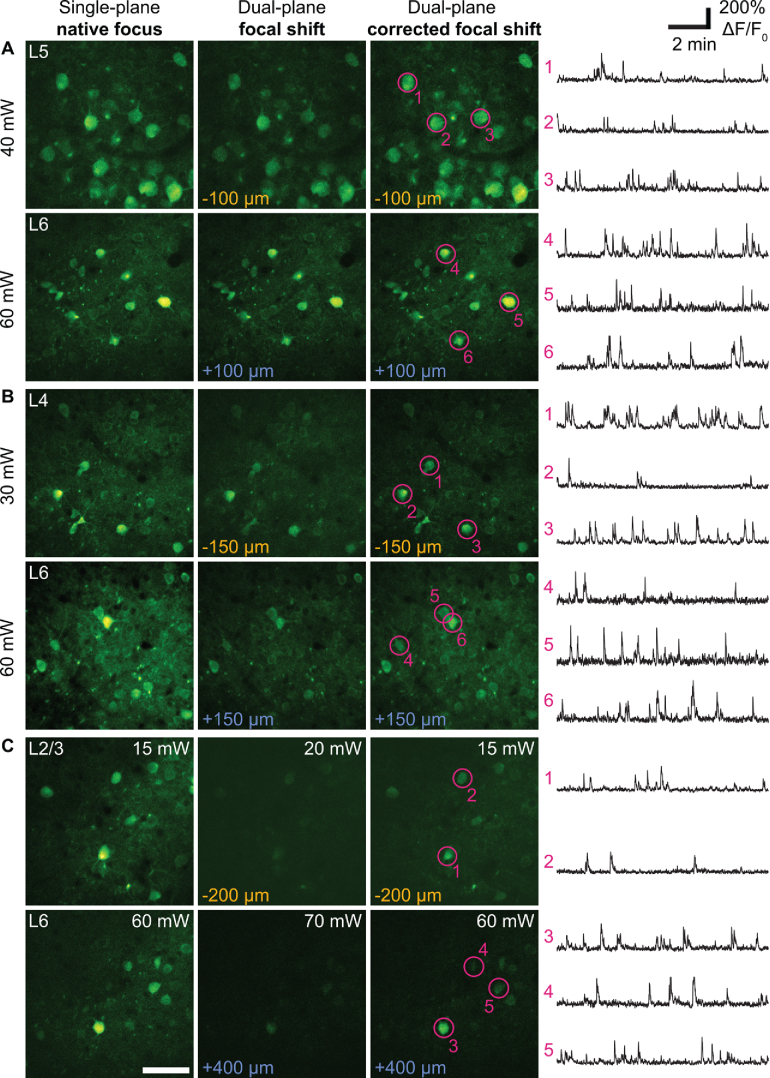
Dual-plane calcium imaging of spontaneous activity in visual cortex. **(A)** L6-L5 acquisition. (Left) Target planes imaged with a single plane native focus acquisition. (Centre) The same planes imaged with focal shifts applied for a dual-plane acquisition. The focal shifts used for each plane are shown in the bottom left of the image, colour indicating whether the acquisition was made with the delayed or non-delayed beam. (Right) The same planes imaged with a dual-plane aberration-corrected acquisition. Example traces are shown to the right for the circled cells. The same panels of images and example traces are shown for a L6-L4 acquisition in **(B)** and for a L6-L2/3 acquisition in **(C)**. All images are averages of 100 frames. The scale bar is 50 µm.

To examine the performance of the remote focus and aberration correction systems during *in vivo* imaging, we performed dual-plane calcium imaging in mouse cortex across a range of axial separations, both with and without applying the DM correction maps collected previously from imaging fluorescent beads. We recorded spontaneous activity from neurons in the primary visual cortex of C57BL/6 mice injected with AAV2/1-Syn-GCaMP7f (1.9 × 10^13^ genome copies per mL). To demonstrate the ability of this system to record from any pair of cortical layers, we made three dual-plane recordings targeted to the following layers: L6 and L5 (Fig. 6(A)), L6 and L4 (Fig. 6(B)), L6 and L2/3 (Fig. 6(C)).

First, we acquired images of each of the target planes with single-plane imaging. This was done with the native focus of iObj while the delayed path was shuttered and a flat map was applied to the non-delayed DM. These images provided references for the degradation of signal quality with focal shift and the recovery of signal quality with aberration correction. Second, the delayed path was unshuttered and the required focal shifts were applied to both beams for a dual-plane acquisition of the target planes. Third, a dual-plane aberration-corrected acquisition was made, during which the two DMs displayed the correction maps for the focal shift applied to their beam. Lastly, a 10-minute dual-plane aberration-corrected acquisition was made at 7 Hz to record calcium activity from the target planes. Acquisitions were then motion-corrected and segmented with Suite2p (version 0.11.1, [50]).

Consistent with our resolution measurements, for small axial separations (< 200 µm) there was little observable difference in signal quality between the three imaging conditions of single-plane, non-corrected dual-plane and aberration-corrected dual-plane imaging (Fig. 6(A), Fig. S1(A,B)). However, since 3p excitation efficiency is proportional to NA^2^ [51], the signal quality of 3p imaging decreases as axial PSF expands. Thus, for larger axial separations, the necessity of aberration-correction to maintain not only single-cell resolution but also signal quality became more apparent (Fig. 6(B,C), Fig. S1 (C,D)). Calcium transients recorded during the maximum axial separation applied *in vivo* (600 µm) were slightly lower in amplitude than those recorded during smaller focal shifts, potentially due to the larger axial PSF introducing stronger contamination from surrounding neuropil (Fig. S2).

## 4. Discussion

This system combines three established microscopy techniques – temporal multiplexing, non-unity magnification remote focusing and sensorless AO – into an assembly which can be retrofit to any existing 3p microscope to bestow the ability to simultaneously image any pair of cortical layers.

The optical design described here was conceived to support experiments exploring the role of L6 in cortical processing. Prior to this work, the only published demonstration of multi-layer imaging which included L6 was produced with a laser source and microscope significantly more expensive and specialised than those most commonly purchased by research groups adopting 3p microscopy and was designed to image an entire cortical column near-simultaneously [44]. While it remains a technical marvel, constructing such a system in order to investigate interlaminar circuits of L6 would be an over-complicated and costly endeavour. Rather, several lines of experimentation would be well served by a more straightforward approach prioritising axial separation between two 3p imaging planes to record simultaneous activity from one deep and one superficial layer.

Improvements in the performance of this system are foreseeable and will likely be heralded by advancements in 3p excitation sources, particularly power output. With greater power output, unity-magnification remote focusing wherein zObj and iObj are identical becomes more feasible as the power sacrifice of a double-pass through a high NA objective becomes less critical. Further, a raised power ceiling would allow additional temporally multiplexed planes, particularly if they lie close (<150 µm) to the native focal plane of the imaging objective and thus have no need for aberration correction.

While all commercial 3p sources provide tunable repetition rates up to 4 MHz, 3p imaging has thus far largely been confined to repetition rates ≤ 1 MHz. This is partly due to long-held concerns that the longer wavelengths and high pulse energies required by 3p excitation [51] would result in thermal tissue damage when combined with faster repetition rates [52,53]. However, in recent years, demonstrations of 3p imaging at 2 MHz have begun to emerge [45,54], alongside calculations predicting a safe range of up to 7–10 MHz for 3p imaging of cortex [55,56]. These calculations assume 3p excitation is sufficiently efficient that imaging the depth of cortex is achievable with pulse energies no higher than 20 nJ at the surface of the brain. If excitation is less efficient, this narrows the safe range of repetition rate. Since 3p excitation efficiency is inversely proportional to the square of the product of pulse width and repetition rate [51], sufficient pulse compression and the avoidance of third-order dispersion are essential steps in the pursuit of 3p imaging at higher repetition rates. If proven workable, the improved sampling frequency of higher repetition rate imaging would allow near-simultaneous volume acquisitions achieved by frame-wise scanning of the imaging objective. This would permit this system to scan each temporally multiplexed beam over multiple near-simultaneous planes, increasing the volume of tissue sampled and extending the axial range.

Lastly, in principle, the AO module incorporated into this system could be used to correct for both the aberrations produced by the system during focal shifts at the same time as the aberrations produced by scattering tissue during *in vivo* imaging. This additional correction would improve imaging quality for all *in vivo* acquisitions and becomes increasingly more valuable the deeper an imaging plane is positioned [47]. Applying the AO module to correct for both system and tissue-induced aberrations would require the system correction map to be applied before tissue aberration measurement could begin. Depending on focal shift applied, a variable portion of the stroke of each of the DM actuators would be devoted to system correction, meaning the capacity of the DM to correct for tissue-induced aberrations would diminish with increasing focal shift.

We present this system as a simpler and lower cost alternative to hybridising 2p-3p excitation for experiments investigating the functional circuitry of structures which lie beyond the depth limit of 2p imaging.

## Supplemental information

Supplement 1 Supplemental Document https://doi.org/10.6084/m9.figshare.27868041

## Data Availability

Data underlying the results presented in this paper are not publicly available at this time but may be obtained from the authors upon reasonable request.

## Supplemental document

See Supplement 1 for supporting content.
