# Supplementary material for: In vivo dual-plane 3-photon microscopy: spanning the depth of the mouse neocortex
Source: Biomed Opt Express. 2024 Nov 26;15(12):7022–34. doi: 10.1364/BOE.544383 (PMC11640578; doi:10.1364/BOE.544383)
Supplement: Supplement 1 [file boe-15-12-7022-s001.pdf]

Supplemental Document

Biomedical Optics EXPRESS

In vivo dual-plane 3-photon microscopy: spanning the depth of the mouse neocortex: supplement

MATILDA CLOVES* **AND TROY W. MARGRIE**

The Sainsbury Wellcome Centre for Circuits and Behaviour, University College London, 25 Howland Street, W1T 4JG, London, United Kingdom
**tilly.cloves.15@ucl.ac.uk*

This supplement published with Optica Publishing Group on 26 November 2024 by The Authors under the terms of the Creative Commons Attribution 4.0 License in the format provided by the authors and unedited. Further distribution of this work must maintain attribution to the author(s) and the published article's title, journal citation, and DOI.

Supplement DOI: https://doi.org/10.6084/m9.figshare.27868041

Parent Article DOI: https://doi.org/10.1364/BOE.544383

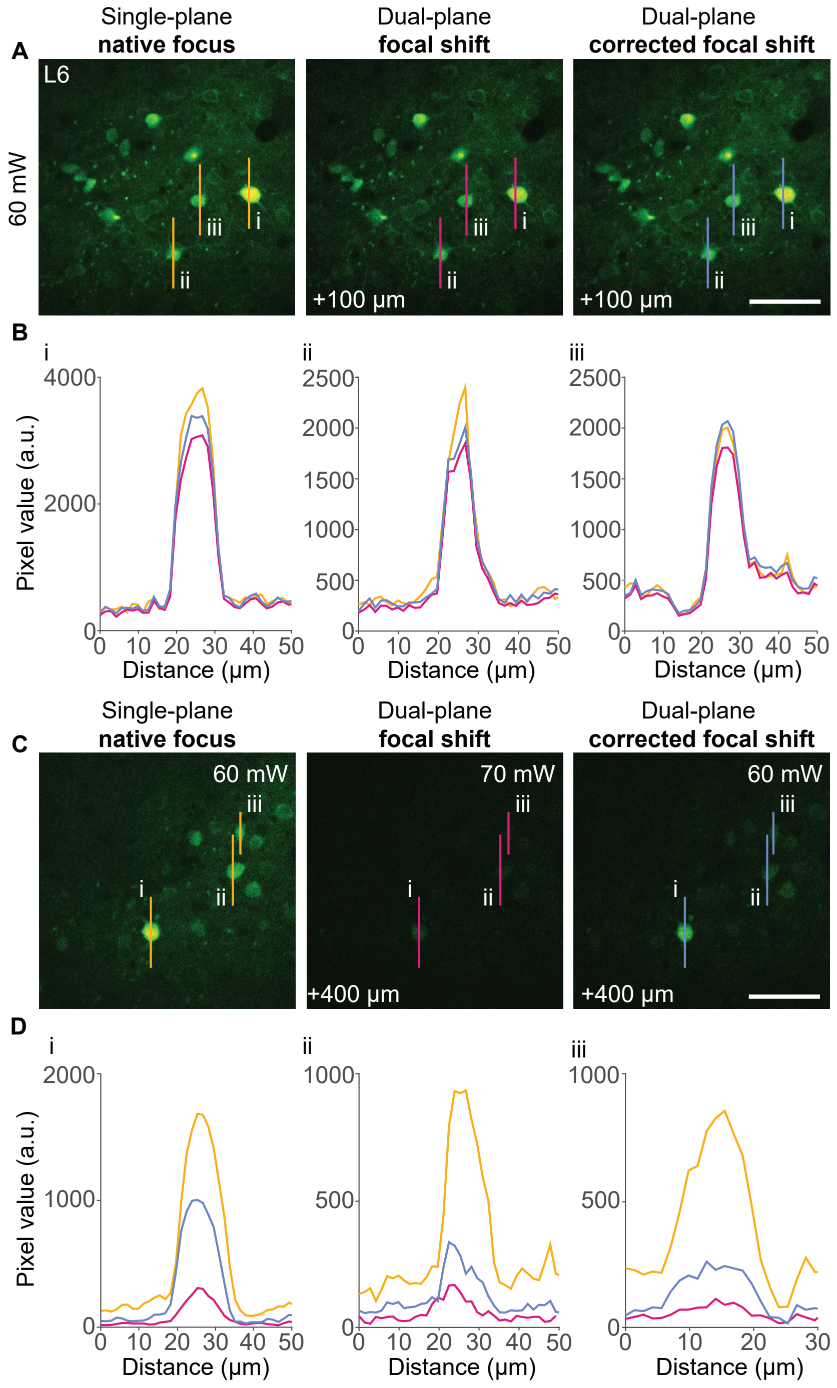

Fig. S1. In vivo 3p excited fluorescence signal is inversely propo rtional to axial resolution and thus also to focal shift. **A** The same L6 images as shown in Fig. 6A. (Left) Target plane imaged with a single plane native focus acquisition. (Centre) The same plane imaged with a +100 µm focal shift applied for a dual-plane acquisition. (Right) The same plane imaged with an aberration-corrected focal shift. All images are averages of 100 frames. The scale bar is 50 µm. **B** Profiles of pixel values along the lines shown in A. **C** As in A for the L6 images shown in Fig. 6C. **D** As in B for the lines shown in C.

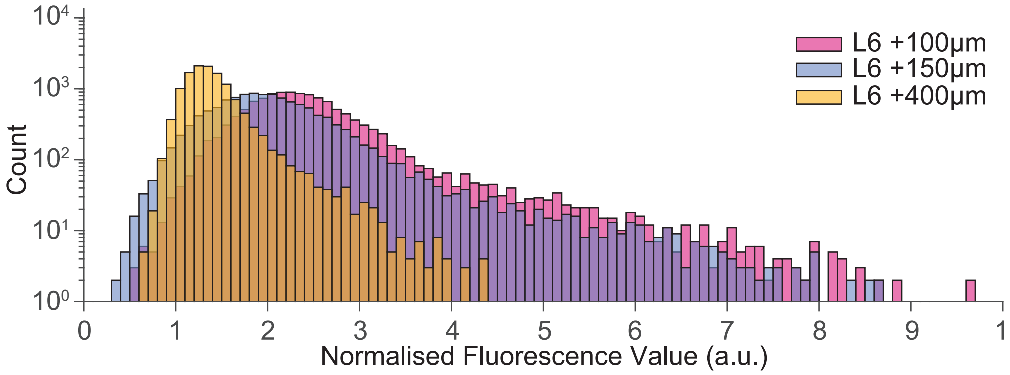

Fig. S2. Amplitudes of calcium transients are inversely proportional to axial resolution and thus also to focal shift. Three histograms show the distributions of neuropil-subtracted fluorescence values collected from all L6 ROIs during the 10-minute recordings of spontaneous activity. The pink histogram shows the distribution of fluorescence values recorded with the +100 µm aberration-corrected focal shift, while the blue shows the same for the +150 µm aberration-corrected focal shift, and the yellow for the +400 µm aberration-corrected focal shift.
